# Notopterol mitigates IL-1β-triggered pyroptosis by blocking NLRP3 inflammasome via the JAK2/NF-kB/hsa-miR-4282 route in osteoarthritis

**DOI:** 10.1016/j.heliyon.2024.e28094

**Published:** 2024-03-13

**Authors:** Ko-Ta Chen, Chi-Tai Yeh, Vijesh Kumar Yadav, Narpati Wesa Pikatan, Iat-Hang Fong, Wei-Hwa Lee, Yen-Shuo Chiu

**Affiliations:** a Department of Orthopedics, Taipei Medical University Hospital, Taipei, 11031, Taiwan; b Department of Medical Research, Shuang Ho Hospital, Taipei Medical University, New Taipei City, 23561, Taiwan; c Continuing Education Program of Food Biotechnology Applications, College of Science and Engineering, National Taitung University, Taitung, 95092, Taiwan; d Department of Pathology, Shuang Ho Hospital, Taipei Medical University, New Taipei City, 23561, Taiwan; e Department of Orthopaedics, School of Medicine, College of Medicine, Taipei Medical University, Taipei, 11031, Taiwan; f Department of Orthopedics, Shuang Ho Hospital, Taipei Medical University, New Taipei City, 23561, Taiwan; g School of Nutrition and Health Sciences, College of Nutrition, Taipei Medical University, Taipei, 11031, Taiwan; h Research Center of Geriatric Nutrition, College of Nutrition, Taipei Medical University, Taipei, 11031, Taiwan

**Keywords:** Osteoarthritis, Notopterol, Interleukin 1 beta, Pyroptosis, NLRP3 inflammasome, JAK2/NF-κB/hsa-miR-4282 pathway

## Abstract

**Objective:**

Osteoarthritis (OA), the most prevalent form of arthritis, impacts approximately 10% of men and 18% of women aged above 60 years. Currently, a complete cure for OA remains elusive, making clinical management challenging. The traditional Chinese herb *Notopterygium incisum*, integral to the Juanbi pill for rheumatism, shows promise in safeguarding chondrocytes through its strong anti-inflammatory effects.

**Methods:**

To explore the protective effect of notopterol and miRNA (has-miR-4248) against inflammation, we simulated an inflammatory environment in chondrocytes cell lines C20A4 and C28/12, focusing on inflammasome formation and pyroptosis.

**Results:**

Our finding indicates notopterol significantly reduced interleukin (IL)-18 and tumor necrosis factor (TNF)-alpha levels in inflamed cells, curtailed reactive oxygen species (ROS) production post-inflammation, and inhibited the JAK2/STAT3 signaling pathway, thus offering chondrocytes protection from inflammation. Importantly, notopterol also hindered inflammasome assembly and pyroptosis by blocking the NF-κB/NLRP3 pathway through hsa-miR-4282 modulation. *In vivo* experiments showed that notopterol treatment markedly decreased Osteoarthritis Research Society International (OARSI) scores in OA mice and boosted hsa-miR-4282 expression compared to control groups.

**Conclusions:**

This study underscores notopterol's potential as a therapeutic agent in OA treatment, highlighting its capacity to shield cartilage from inflammation-induced damage, particularly by preventing pyroptosis.

## Highlights

• Notopterol Inhibits Structural Damage in Osteoarthritis.
• JAK2/NF-κB/miR-4282 signaling in articular chondrocytes development.
• Notopterol Suppresses expression of (ii) JAK2/NF-κB and an increased level of miR-4282.

## 1 Introduction

Osteoarthritis (OA), the most prevalent joint disease globally, affects nearly 10% of men and 18% of women over 60. OA poses a significant threat to the quality of life and is projected to be the primary cause of global workforce disability by 2030 [1]. This progressive condition can lead to debilitating pain and functional impairment, significantly impacting socio-economic well-being in developed nations [2]. Although end-stage OA is often managed with pain medications and joint replacements, these approaches overlook the early disease-associated morbidity and potential complications related to surgery, such as risks associated with adverse prognosis and the limited lifespan of the prosthesis [3]. Despite the widespread occurrence of OA, current treatment strategies remain insufficient and managing the disease presents considerable challenges. Non-surgical therapies to delay deterioration and alleviate pain have been explored extensively, yet arthroplasty often emerges as the only surgical solution for severe OA cases unresponsive to conservative treatments [4,5]. Its progression is a complex process involving an intricate interplay of numerous mechanisms, as evidenced by comprehensive studies and clinical observations. Both mechanical and biological factors contribute to the advancement of the disease, with immune responses playing a central role in the degradation of joint structures. Two key proinflammatory cytokines, tumor necrosis factor-alpha (TNF-α) and interleukin-1 beta (IL-1β) are widely recognized as the drivers of OA pathology. These cytokines are secreted by a range of cell types involved in joint health, including immune cells (macrophages), cartilage cells (chondrocytes), bone cells (osteoprogenitors, osteoblasts, osteoclasts, and osteocytes), and cells that line the joint capsule (synovial fibroblasts) [6,7]. In OA, the levels of TNF-α and IL-1β are markedly elevated in the synovial fluid, the lubricating fluid found in the joint space, as compared to healthy joints. Upon induction by IL-1β and TNF-α, these cells release a spectrum of inflammatory proteins and enzymes, including aggrecanases and matrix metalloproteases (MMPs; specifically, MMP-3, MMP-7, and MMP-13). These enzymes are instrumental in the breakdown of cartilage, the protective tissue at the ends of bones that ensures smooth joint movement. They degrade aggrecan and collagen type II, the primary constituents of cartilage [[8], [9], [10]].

The pathogenesis of OA is also extensively influenced by the Janus Kinase-Signal Transducer and Activator of Transcription (JAK-STAT) signaling pathway [11]. This pathways is essential for transmitting extracellular signals, triggered by cytokines and growth factors, to the nucleus, causing the expression of target genes. In the context of OA, the JAK-STAT pathway, particularly the JAK2/STAT3 signaling axis, has been implicated in the disease's progression [12]. It influences the inflammatory response, chondrocyte metabolism, and cartilage degradation. Activation of this pathway can enhance the production of matrix-degrading enzymes and inflammatory cytokines, leading to cartilage extracellular matrix degradation and synovial inflammation, key hallmarks of OA [13]. Given its pivotal role in OA pathogenesis, the JAK-STAT pathway has become a significant therapeutic target [14]. An alternative noteworthy feature of programmed cell death involves cellular swelling and the release of pro-inflammatory substances, a process known as pyroptosis, which results in an inflammatory response in the nearby environment in OA [15]. Unlike apoptosis, a form of cell death that usually occurs without eliciting an immune response, pyroptosis is highly pro-inflammatory and can, therefore, notably impact the progression of inflammatory diseases such as OA [16].

In the context of OA, the role of pyroptosis becomes especially crucial in chondrocytes - cells responsible for producing and maintaining the cartilage matrix [17]. These cells are pivotal to joint health, and their degradation can lead to the clinical manifestations of OA, including joint pain, stiffness, and loss of function [18]. When subjected to various stressors, chondrocytes can undergo pyroptosis, leading to the release of potent pro-inflammatory cytokines and other substances. This resultant pro-inflammatory environment accelerates cartilage degradation and incites further inflammation within the joint, thereby exacerbating the symptoms of OA [19]. As such, understanding the processes and pathways involved in chondrocyte pyroptosis can provide invaluable insight into the pathogenesis of OA, and potentially open avenues for novel therapeutic strategies to prevent or reduce the disease progression. Pyroptosis in OA contributes to the release of pro-inflammatory cytokines and other substances that further fuel cartilage destruction and joint inflammation, intensifying its symptoms. A critical component involved in pyroptosis is the inflammasome, a multi-protein complex that regulates the activation of inflammatory responses [20]. The NLRP3 inflammasome, a specific subtype of the inflammasome family, has gained considerable attention in OA research [21]. The NLRP3 inflammasome is activated by cellular stress signals, such as DAMPs like uric acid crystals and matrix fragments. Its activation leads to the release of IL-1β and IL-8, fueling the inflammatory response and contributing to cartilage degradation in OA [22].

Moreover, non-coding RNA molecules, such as microRNAs (miRNAs) which are crucial for gene regulation, plays vital role in various cellular process, such as cell proliferation, differentiation, and apoptosis [23]. Their contribution to the pathogenesis of OA is becoming increasingly recognized, particularly in terms of influencing inflammation and matrix degradation [24]. Notably, miR-21-5p emerges as a key player in OA, potentially altering the disease course by promoting hyaline cartilage production [25]. miRNAs also regulate inflammatory responses in chondrocytes, contributing to the prevalent inflammation in OA [25]. They affect critical pathways in OA progression, such as JAK-STAT signalling pathways, influencing inflammatory cytokine production and chondrocyte pyroptosis [26]. Consequently, miRNAs offer a promising therapeutic target in OA, warranting further research to elucidate their specific roles and interactions with various signaling pathways in the disease.

The role of natural compounds in protecting OA has gained significant attention in recent years. These compounds, derived from various natural sources such as plants, herbs, and fruits, have shown promising potential in mitigating OA progression and providing therapeutic benefits [27,28]. *Notopterygium incisum* is a species of Notopterygium, a traditional Chinese medicinal plant that is used to make the Juanbi pill, which is used for the treatment of rheumatism in conjunction with other herbs. Following therapy with the herb, a mass spectrometry test of the patient's blood serum has shown that notopterol is one of the most prevalent enriched components in blood [29,30]. Notopterol may bind to three key locations in the kinase domains of JAK2 and JAK3 (L932/R980/N981 and K830/L905/D967; respectively) to suppress the JAK-STAT signaling axis, resulting in reduced secretion of inflammatory cytokines and chemokines, according to the prior investigation. This work suggests that the notopterol could be used to manage JAK-STAT–related illnesses other than RA [29].

We utilized an *in vitro* approach that includes chondrocyte cell lines, stimulated with the pro-inflammatory cytokine IL-1 to simulate a pro-inflammatory milieu in OA. Then, notopterol was used to treat these stimulated cells at various doses. We examined the effects of notopterol on chondrocyte reactive oxygen species (ROS) generation and inflammasome formation. Furthermore, we have established the mechanism of notopterol-mediated inflammation inhibition through the evaluation of its effects on the JAK2/STAT3 pathway and the involvement of microRNA-aiming nuclear factor kappa B (NF-κB)/NLR family, pyrin domain containing 3 (NLRP3) pathways. Finally, we demonstrated the anti-inflammatory effect of the notopterol by using an *in-vivo* OA animal model.

## 2 Materials and methods

### 2.1 Cells, culture medium and reagents

Human C20A4 and C28/I2 chondrocytes were cultured in a medium comprising an equal combination of Dulbecco's Modified Eagle's Medium (DMEM) and F12 medium, both procured from GIBCO, Life Technologies Corp., Carlsbad, CA, USA. This mixture was fortified with 10% fetal bovine serum (FBS), likewise acquired from GIBCO, Life Technologies Corp., Carlsbad, CA, USA. The culture medium was renewed every three days to facilitate growth until the cells achieved full confluence, typically necessitating subculturing at intervals of 4–5 days. To ensure experimental consistency, we utilized cells at the same passage level and routinely performed checks to confirm the cell line's identity and to detect any potential contamination. Notopterol, with a purity of 98% or higher, was procured from Selleck Chemicals in Houston, TX, USA, and prepared as a 50 mM stock solution in DMSO.

### 2.2 Sulforhodamine B assay

Chondrocyte C20A4 and C28I2 cells were plated in 96-well plates with a density of 3000 cells per well. Twenty-four hours later, once the cells had adhered, they were divided into control and treatment groups. The treatment group received various concentrations of notopterol, while the control group was treated with dimethyl sulfoxide (DMSO). After 48 h of incubation, the medium was removed, and each well was treated with 100 μL of 10% trichloroacetic acid (TCA). The TCA was removed after a 1-h incubation at 4 °C, and then 100 L of SRB reagent was added to each well, which was then incubated for 1 h at room temperature. The wells were washed with 1% acetic acid. Once the plates had been dried for 20 min at 60 °C, 200 μL of 20 mM Tris buffer was added to each well. Absorbance at 565 nm was measured using spectrophotometry to assess cell viability, with the results being compared between the treatment and control groups to calculate the percentage of viable cells.

### 2.3 Total RNA extraction, qRT-PCR-mRNA/miRNA quantification, and transfection

Following the manufacturer's guidelines, total RNA was extracted and purified from cell and tissue samples after treatment using a TRIzol-based method (Life Technologies) following the manufacturer's guidelines. One microgram of this RNA was then reverse transcribed with the QIAGEN One-step RT-PCR Kit (QIAGEN, Taiwan), and PCR amplification was conducted using the Rotor-Gene SYBR Green PCR Kit (QIAGEN, Taiwan). Primers for all studied genes were acquired from QIAGEN (Taiwan). For miRNA analysis, RNA was isolated using Trizol (Invitrogen, Life Technologies), and first-strand cDNA synthesis was performed using the PrimeScript RT Reagents Kit (Takara) as per the manufacturer's protocol. The relative levels of miRNA in the samples were determined by qRT-PCR, using U6 miRNA as the standardization control. This process utilized the SYBR Premix Ex Taq Kit (Takara) on a QIAGEN rotor real-time PCR system (QIAGEN, Valencia, CA, USA). Primers for miR-4282 amplification were 5′-AGGATGATGTTCCTGGATGC-3’ (forward) and 5′-GGTGAAGTTCCAGGGGAAGAT-3’ (reverse), with U6 primers being 5′-ATTGGAACGATACAGAGAAGATT-3’ (forward) and 5′-GGAACGCTTCACGAATTTG-3’ (reverse). To modulate miR-4282 expression, cells were transfected with either a miR-4282 mimic or inhibitor (both from Sigma, St. Louis, MO, USA) using Lipofectamine 2000 (Invitrogen, USA) according to the supplier's instructions.

### 2.4 Luciferase reporter assays

To explore the interaction between miR-4284 and its potential target genes NF-κB and NLRP3, miRNA target gene prediction and Luciferase reporter assays were conducted. The specific binding sites for miR-4284 on the 3′-UTR regions of NF-κB and NLRP3 mRNA were identified using the microRNA database (http://www.microma.org/). These predicted sites, along with their respective mutant versions, were PCR-amplified and cloned into the pMIR-GLOTM Luciferase vector (Promega Corporation, Madison, WI, USA) at the *Xho*I and *Sac*I sites, a process initiated by GenePharma. In the Dual-Luciferase assay, cells plated in 96-well formats were co-transfected with either the Pmir-GLO–NF–κB or NLRP-3-3′UTR Luciferase construct containing the anticipated or mutant sequences and 50 nM of miR-4284 mimics or a negative control. Luciferase activity was assessed 24 h post-transfection using the dual-luciferase reporter assay system (Promega., Ltd), with the results being adjusted for Renilla Luciferase activity to obtain relative luciferase activities.

### 2.5 Western blot analysis and cytokine detection

Protein extracts (40 ng) from chondrocytes were electrophoresed on 10% SDS-PAGE and then transferred onto PVDF membranes (Bio-Rad, USA) for 1–2 h at a constant 300 mA. The membranes were blocked using 5% skim milk before being incubated overnight at 4 °C with primary antibodies targeting collagenase type II, JAK2, phosphorylated JAK2, MMP-13, STAT3, phosphorylated STAT3, β-actin, NF-κB, phosphorylated ASC1, and Cas-1, all diluted at 1:1000. Subsequently, appropriate secondary antibodies (refer to Supplementary Table S1) were applied at room temperature for 2 h. The detection of the protein bands was achieved using an Enhanced Chemiluminescence kit, and band intensities were analyzed using Image Lab 3.0 software (Bio-Rad). Additionally, the presence of inflammatory cytokines in the cell culture supernatants was assessed. The levels of these cytokines were quantified using ELISA kits for mouse TNF-α (EK0527), IL-6 (EK0411), and MMP9 (A00420-2) from Boster Biological Technology.

### 2.6 Immunofluorescence

After a 48-h incubation on coverslips, chondrocytes were fixed using a 4% paraformaldehyde solution provided by Santa Cruz Biotechnology, USA. They were then incubated in a blocking solution containing 0.1% Triton X-100 (sourced from Sigma Aldrich, Germany) in PBS solution Overnight incubation at 4 °C followed, during which the samples were treated with polyclonal antibodies anti-Rat NLRP3, NF-κB, *p*-JAK2, or N-GDSMD at a dilution of 1:60 (Abcam, USA). After thrice washing with PBS, the specimens were incubated at 37 °C for 1 h with an anti-mouse IgG secondary antibody at a dilution of 1:200 (Abcam, USA) and DAPI (acquired from Roche Applied Science, Germany) for nuclear staining. The resulting images were captured using an Olympus FluoView FV10i confocal microscope.

### 2.7 Establishment of the mice OA model

Male C57BL/6 mice (n = 45, aged 10 weeks), free of pathogens, were obtained from BioLASCO Taiwan Co. Ltd, in accordance with the protocols sanctioned by the Laboratory Animal Committee of Taipei Medical University (protocol number LAC-2020-0146). These mice were randomly distributed into three distinct groups, with each group comprising 15 mice: (1) a sham group, (2) an osteoarthritis (OA) group that received intraperitoneal injections of DMSO, and (3) OA group treated with notopterol also through intraperitoneal injections. To induce OA, a surgical procedure creating instability of the medial meniscus (DMM) was performed, as previously detailed. The procedure involved severing the right knee joint capsule adjacent to the medial patellar tendon and detaching the medial meniscus ligament with microsurgical scissors in the OA and OA + notopterol groups. For the sham group, the joint capsule was incised without damaging the medial meniscus ligament. Eight weeks post-surgery, the mice were euthanized, and their knee joints were harvested for histological analysis.

### 2.8 Analysis of tissue histology

Initially, knee joints were fixed at 37 °C for 48 h in 4% paraformaldehyde, followed by decalcification in a 10% EDTA solution at room temperature for four weeks, respectively. After decalcification, the samples were embedded in paraffin and sectioned into 6-μm thick slices. Sections from each group (sham, OA, and OA + Notopterol) were then stained using the Solarbio Modified Safranine O-Fast Green FCF Cartilage Stain kit. Histomorphometric analysis was performed on 15 mice per group, utilizing the Osteoarthritis Research Society International (OARSI) scoring system to assess cartilage degradation. This system, which involves a blinded examination of the tissue slices under a microscope, includes 6 grades and 4 stages, with total scores ranging from 0 to 24 based on the extent of OA and cartilage damage, as detailed in Supplementary Table S2. The scoring criteria were as follows: Grade 0 indicates intact surface and cartilage; Grade 1, an intact surface; Grade 2, surface discontinuity; Grade 3, vertical fissures; Grade 4, erosion; Grade 5, denudation; and Grade 6, deformation. The severity of cartilage degeneration increases with the score. Additionally, the thickness of the medial subchondral bone plate was measured in both HE and Safranin O-stained sections using Axio Vision software. Cartilage evaluation and scoring were primarily conducted by independent investigators not involved in the study.

### 2.9 Statistical analysis

All experiments were conducted thrice in triplicates. The results are shown as the mean and standard deviation, with a p-value of 0.05 indicating statistical significance. The analysis of Western blotting, migration, and invasion assays was performed using ImageJ (version 1.51j8), while the SRB assay analysis was carried out with GraphPad Prism (version 8.00). Both ImageJ (version 1.51j8) and GraphPad Prism (version 8.00) were utilized to quantify the outcomes of the *in vivo* studies.

## 3 Results

### 3.1 Notopterol reduces IL-18 and TNF-α levels of IL-1β–induced inflammation in C20A4 and C28I2 cells

**Fig. 1:**
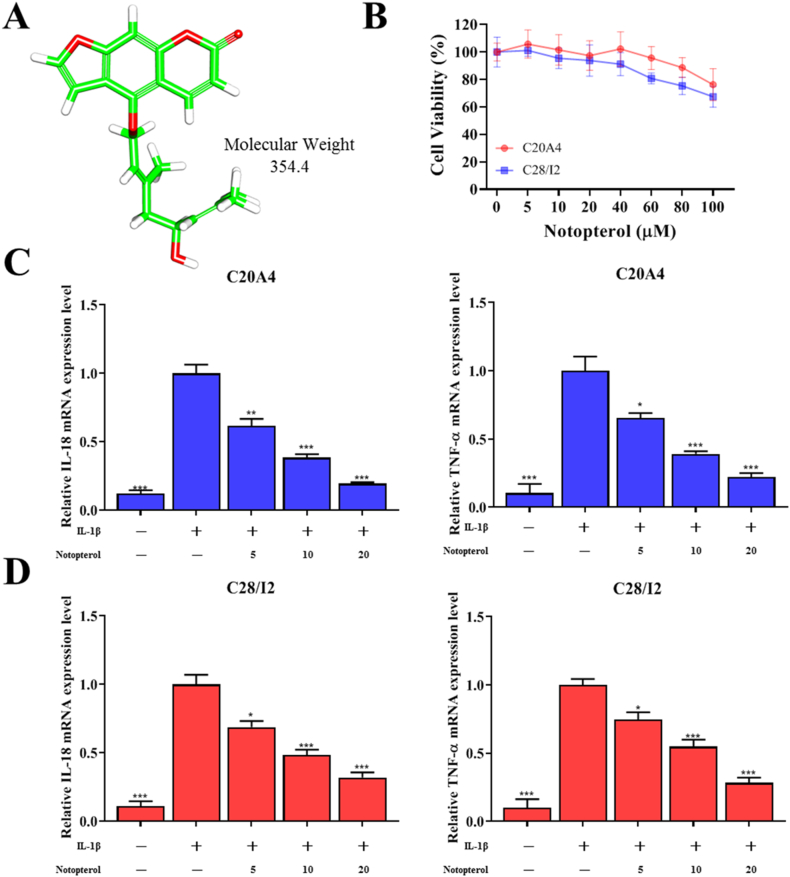
**Notopterol attenuated IL-1β–induced inflammatory environment in chondrocytes.** (**A**) Chemical structure of Notopterol. (**B**) Chondrocytes were treated with Notopterol at different doses (5, 10, 20, 40, 60, 80, and 100 μM) for 48 h. (**C**) C20A4 and (**D**) C28I2 cells were treated with IL-1β (10 ng/mL) and 5, 10, or 20 μM of Notopterol. IL-18 and TNF-α levels were evaluated. Values are expressed as the mean ± standard error of the mean (n = 3). **P < 0.01 and ***P < 0.001 versus the IL-1β-only treatment group.

To determine the anti-inflammatory role of notopterol (structure, Fig. 1A), we first evaluated the effect of notopterol on C20A4 and C28I2 cell lines. Using the SRB assay, we demonstrated that the administration of 0–100 μM notopterol is not toxic to cells and did not significantly change the cell viability (Fig. 1B). We incubated C20A4 and C28I2 cell lines with IL-1 (10 ng/mL) to simulate the pro-inflammatory environment in OA. IL-1β treatment increases the inflammatory cytokines IL-18 and TNF-α expression levels. However, notopterol indicated a dose-dependent relationship with IL-18 and TNF-α mRNA levels in C20A4 (Fig. 1C) and C28I2 cell lines (Fig. 1D).

### 3.2 Notopterol reduces ROS generation on IL-1β–treated chondrocytes

**Fig. 2:**
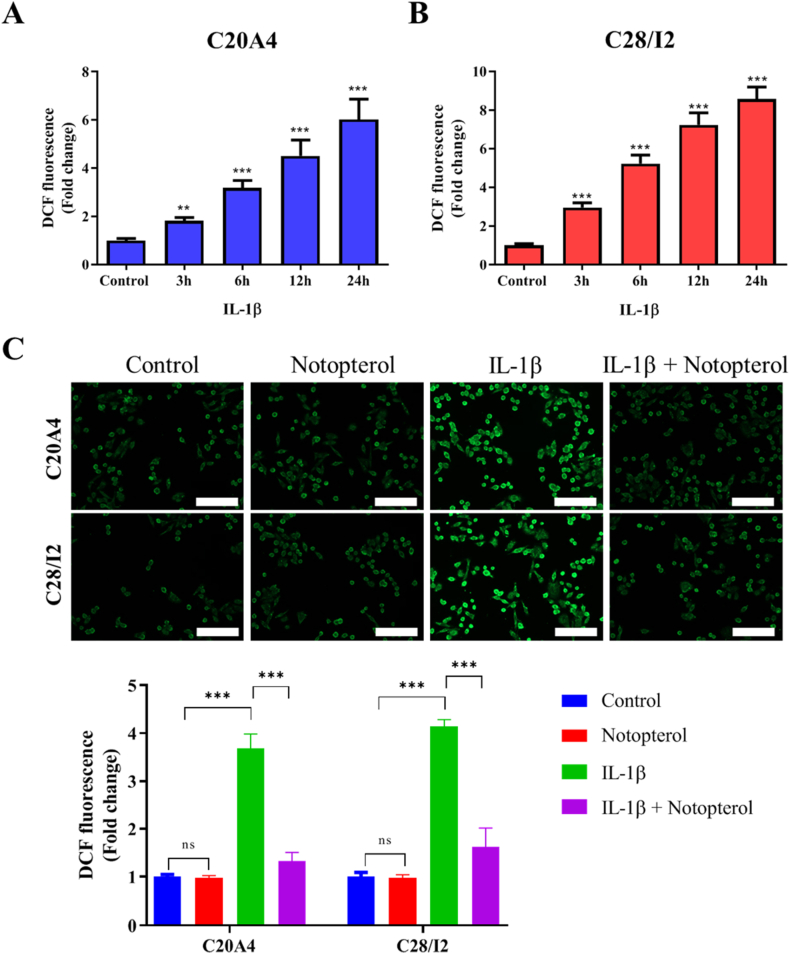
**Notopterol attenuated ROS generation in chondrocytes.** (**A, B**) Chondrocyte cell lines prelabeled with 10 μM DCFH2-DA were exposed to 10 ng/mL IL-1β with increasing time points (3, 6, 12, and 24 h). (**C**) Representative image and quantification of DCF fluorescence assay of C20A4 and C28I2 cell lines treated with IL-1β and Notopterol (20 μM) for 48 h. Fluorescence signals corresponding to ROS levels were recorded at excitation/emission of 485 nm/528 nm. Values are expressed as the mean ± standard error of the mean (n = 3). **P < 0.01 and ***P < 0.001.

Inflammation is strongly associated with ROS generation. Hence, we determined the effect of IL-1 (10 ng/mL) on ROS generation at various times. As expected, IL-1β treatment significantly increased DCF fluorescence, which translates into high ROS levels, in a time-dependent manner (Fig. 2A & B). We then treated the cells with notopterol and evaluated the mechanism by which it may affect ROS generation. Notopterol significantly reduced the DCF fluorescence of C20A4 and C28I2 cell lines treated with IL-1β (Fig. 2C).

### 3.3 Notopterol protects cartilage degradation by inhibiting JAK2/STAT3 and NF-κB signaling

**Fig. 3:**
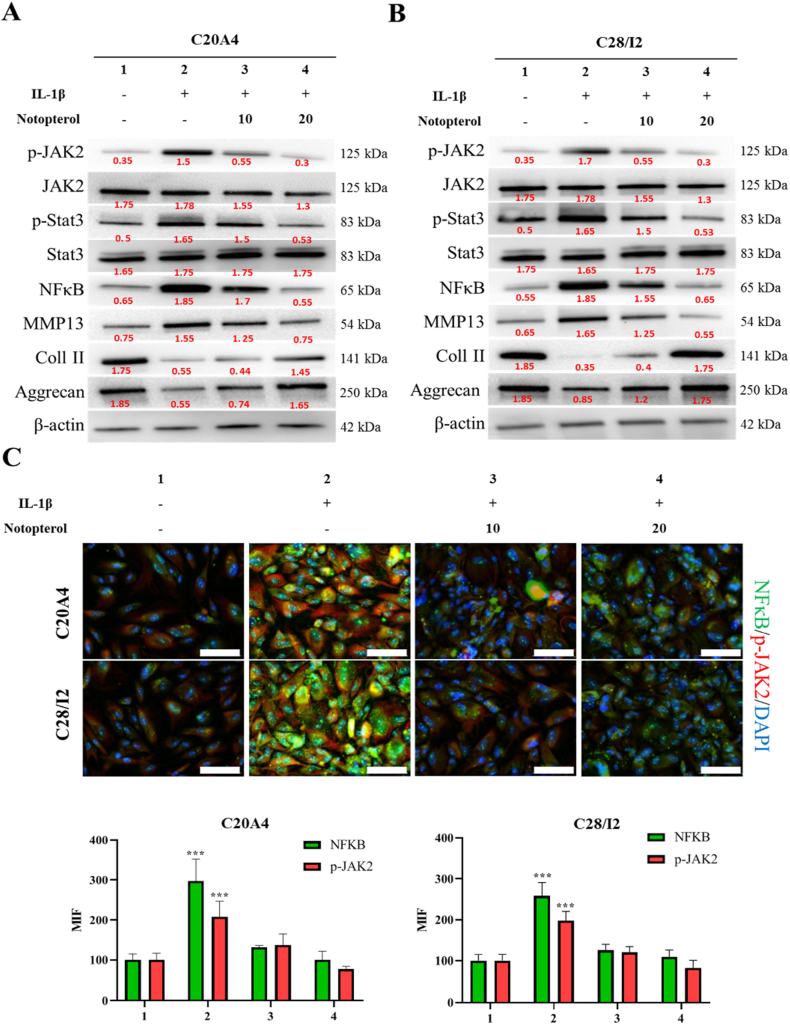
**Effects of notopterol treatment on inflammation-associated protein expression.** Protein expression of *p*-JAK2, JAK2, p-STAT3, STAT3, NF-κB, MMP13, Col II, and aggrecan isolated from (**A**) C20A4 and (**B**) C28I2 cell lines untreated and treated with IL-1β only or combined with either 10 or 20 μM Notopterol were evaluated. Cells were lysed and protein content was quantified and subsequently evaluated using Western blot assay. (**C**) Cells were treated with IL-1β only or in combination with either 10 or 20 μM Notopterol. Cells were incubated with primary antibodies overnight and then with their appropriate secondary antibodies. The expression of *p*-JAK2 (red) and NF-κB (green) overlaid with DAPI (blue) was then observed and quantified. Values are expressed as the mean ± standard error of the mean (n = 3). **P < 0.01 and ***P < 0.001. (For interpretation of the references to color in this figure legend, the reader is referred to the Web version of this article.)

Wang et al. (29). demonstrated that notopterol may bind to three kinase sites in JAK2 in arthritis; we hypothesized that notopterol may exert a similar inhibiting effect on OA. We utilized Western blot analysis to assess the effects of IL-1β–induced inflammation alone or with either 10 or 20 M notopterol on the JAK2/STAT3 signaling pathway. Similar to previous results, notopterol decreased *p*-JAK2 and p-STAT3 expression in a dose-dependent manner. As NF-κB is a major player in the inflammatory response, we also evaluated the NF-κB expression level. Notopterol treatment demonstrated a similar effect on NF-κB expression, where it decreased the NF–B expression level in a dose-dependent way.Similar findings were detected in our evaluation of the cartilage degradation protease MMP-13 and the preservation of cartilage matrix components, namely type II collagen (Col II) and aggrecan, after treatment with either 10 or 20 μM notopterol (Fig. 3A & B). We further validated this finding through immunofluorescence staining of NF-κB and *p*-JAK2. Similarly, IL-1β treatment has increased the intensity of NF-κB and *p*-JAK2 staining, whereas notopterol additionally attenuated the staining intensity of both proteins (Fig. 3C). These experiments demonstrated the notopterol's ability to suppress JAK2/STAT3 and NF–B signaling pathways and may have a protective effect on cartilage cells.

### 3.4 Notopterol suppresses NF-κB-mediated inflammasome and pyroptosis formation

**Fig. 4:**
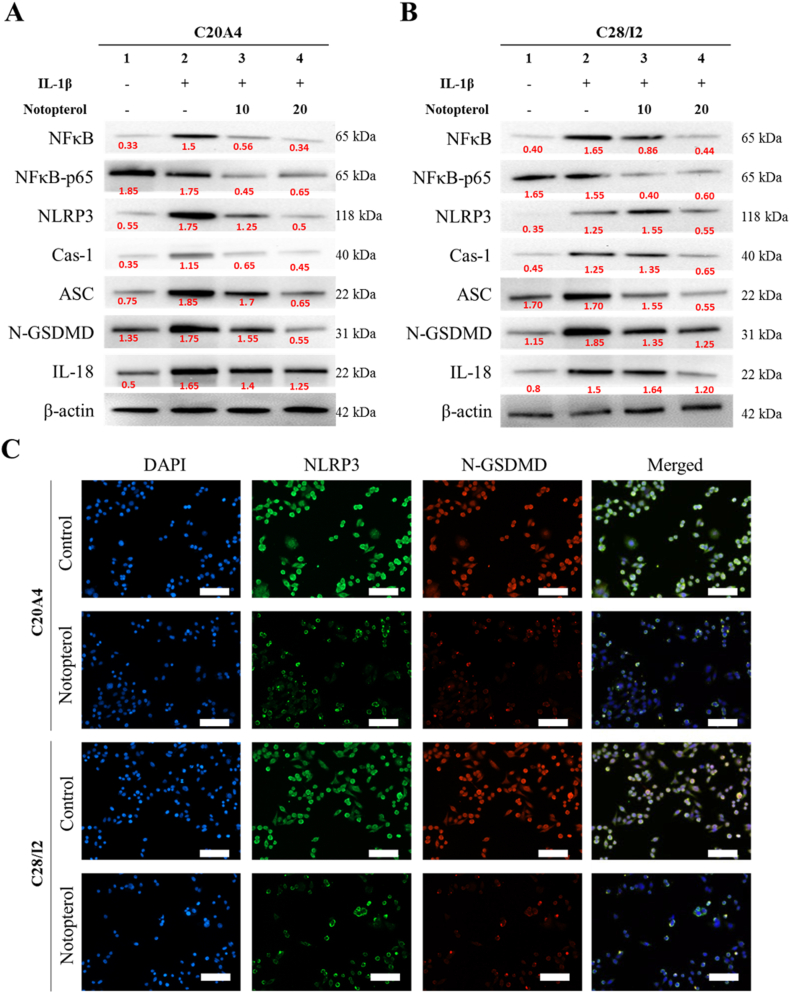
**Notopterol reduced the expression level of inflammasome and pyroptosis-associated protein in human chondrocyte cells.** (**A**) C20A4 and (**B**) C28I2 cells were treated with IL-1β only or combined with either 10 or 20 μM Notopterol for 48 h. Cells were lysed and had their protein isolated and then incubated with primary antibodies for NF-κB, NLRP3, Cas-1, ASC, N-GSDMD, and IL-18. Protein membranes were then incubated with appropriate secondary antibodies and immunoblots were visualized using UVP machine. (**C**) Notopterol attenuated NLRP3-mediated inflammasome formation and pyroptosis marker N-GSDMD. Representative images from an immunofluorescence study showing NLRP3 inflammasome and N-GSDMD expression depicting pyroptosis in C20A4 and C28I2 cell lines. Control cells were treated with IL-1β only and compared with cell lines treated with IL-1β and 20 μM Notopterol. Cells were incubated with primary antibodies overnight and subsequently incubated with their respective secondary antibodies.

As previously stated, inflammation is the major player in OA. Thus, pyroptosis in OA is inevitable, as shown in various OA studies. We suspected that the potent anti-inflammatory activity of notopterol may strongly suppress inflammasome formation and pyroptosis occurrence and alleviate cartilage injury. Furthermore, the IL-1β–induced inflammatory environment increased NF-κB signaling. This, in turn, augmented the inflammasome components NLRP3, Cas-1, and ASC. Pyroptosis occurrence was determined based on the upregulation of N-GSDMD and IL-18. However, treatment with notopterol negatively impacted NF–B-NLRP3-mediated inflammasome formation and pyroptosis in a dose-dependent manner (Fig. 4A & **4B**). We further demonstrated the potential of notopterol through the visualization of the inflammasome formation (NLRP3) and pyroptosis activity (N-GSDMD) of C20A4 and C28I2 cells with immunofluorescence. As expected, notopterol significantly reduced NLRP3 and N-GSDMD expression (Fig. 4C). These data further support the potential use of notopterol in OA treatment.

### 3.5 Notopterol stimulates the expression of has-miR-4282 to reduce NF-κB and NLRP3 levels

**Fig. 5:**
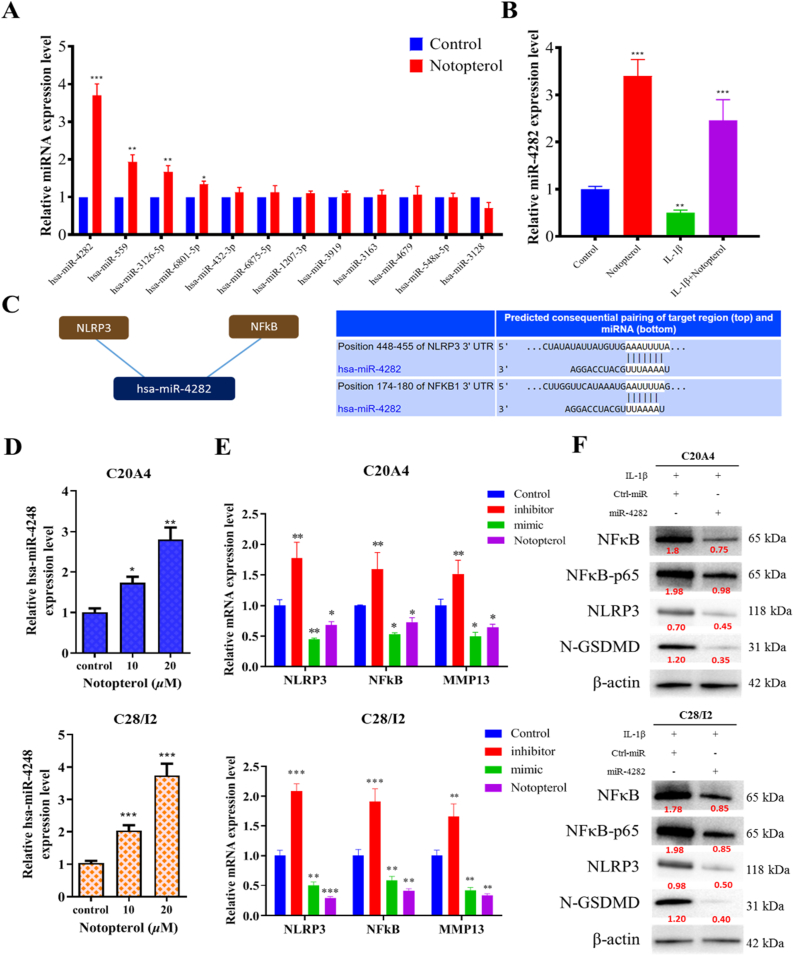
**Notopterol treatment stimulates the expression of hsa-miR-4282 and inhibits the expression of NLRP3 and NF-κB.** (**A**) Notopterol was used to treat the C20A4 cell line and the fold change of 12 predicted miRNAs targeting NLRP3 and NF-κB was observed. **(B)** Relative hsa-miR-4282 miRNA expression of C20A4 cell line treated with IL-1β and Notopterol (20 μM) for 48 h. (**C**) miRNA profiling platform, Targetscan, predicted NLRP3 and NF-κB as hsa-miR-4282 targets. (**D**) Relative hsa-miR-4282 miRNA expression in both C20A4 and C28I2 cell lines was measured after treatment with Notopterol (10 and 20 μM). (**E**) Relative NLRP3 and NF-κB mRNA expression in C20A4 and C28I2 cells were measured after transfection with either hsa-miR-4282 mimic or inhibitor or treatment with notopterol (20 μM). (**F**) Western blot analyses were conducted to evaluate the expression levels of pro-inflammatory marker NF-κB, inflammasome component NLRP3, and pyroptosis marker N-GSDMD. These analyses were performed under the influence of Notopetrol and IL-1β stimulation in cells transfected with miR-4282 and control. The results are presented as mean ± standard error of the mean (n = 3), and statistical significance was determined as **P < 0.01 and ***P < 0.001.

We examined epigenetic factors that may have caused the anti-inflammatory activity of notopterol. Using the TargetScan (http://www.targetscan.org/), an online microRNA (miRNA) profiling platform. We examined the miRNAs with the higher probability of targeting both NLRP3 and NF-κB based on their strong predictive for interaction, broad conservation, and strong complementarity between their 5′ends untranslated region (UTR) and the 3′ ends UTR of NLRP3 and NF-κB mRNA is based on their strong predictive for interaction, broad conservation, and strong complementarity. We incubated the C20A4 cell line with notopterol for 48 h and observed the expression of these miRNAs. From this experiment, we observed that hsa-miR-4282 was significantly altered (Fig. 5A). Notopterol significantly induced the hsa-miR-4282 of C20A4 cell lines treated with IL-1β (Fig. 5B). To validate the prediction that hsa-miR-4282 targets both NLRP3 and NF-κB (Fig. 5C), we designed experiments to investigate the effects of an hsa-miR-4282 mimic and inhibitor and the effect of notopterol administration on hsa-miR-4282 expression. First, we validated the effect of notopterol on hsa-miR-4282 in both C20A4 and C28I2 cell lines. These experimental results revealed a dose-dependent relationship between notopterol and hsa-miR-4282 (Fig. 5D). In the next experiment, we validated NLRP3 and NF-κB as hsa-miR-4282 targets. We transfected either the hsa-miR-4282 mimic or inhibitor to both C20A4 and C28I2 cell lines. We demonstrated that the expression levels of NLRP3 and NF-κB were downregulated with the transfection of the hsa-miR-4282 mimic and were upregulated with the transfection of the hsa-miR-4282 inhibitor. Furthermore, notopterol treatment affected the expression of NLRP3 and NF-κB in the same manner as the hsa-miR-4282 mimic (Fig. 5E). The intriguing aspect of hsa-miR-4248's involvement in pyroptosis and inflammatory cytokine production in IL-1β-stimulated chondrocytes was further highlighted by the combined administration of miR-4248 mimic and notopterol. As depicted in Fig. 5F, the protective effect of hsa-miR-4248 and notopterol was evident through Western blot analyses. These findings demonstrated that the combination of hsa-miR-4248 and notopterol effectively counteracted the impact of IL-1β, resulting in the inhibition of inflammatory responses (including reduced expression of pro-inflammatory and inflammasome markers) and the suppression of pyroptosis in the cells.

Additionally, to show the direct interaction between the molecules, a dual-luciferase reporter assay was conducted. As illustrated in Supplementary Fig. S1, co-transfection with miR-4248 led to a reduction in luciferase activity of the plasmid containing the wild-type fragment of NF-κB or NLRP3 3′-UTR. However, the luciferase activity of the plasmid carrying the mutant NF–B or NLRP3 3′-UTR fragment was not affected by the co-transfection with miR-4248 mimics or negative control. These findings strongly indicated that miR-4248 directly interacted with the 3′-UTR of NF-κB or NLRP3 mRNA. Collectively, this series of experiments provided evidence that notopterol manipulated the expression of NLRP3 and NF-κB through hsa-miR-4282 modulation in C20A4 and C28I2 cell lines.

### 3.6 In-vivo effect of notopterol on cartilage regeneration in an osteoarthritic mouse model

**Fig. 6:**
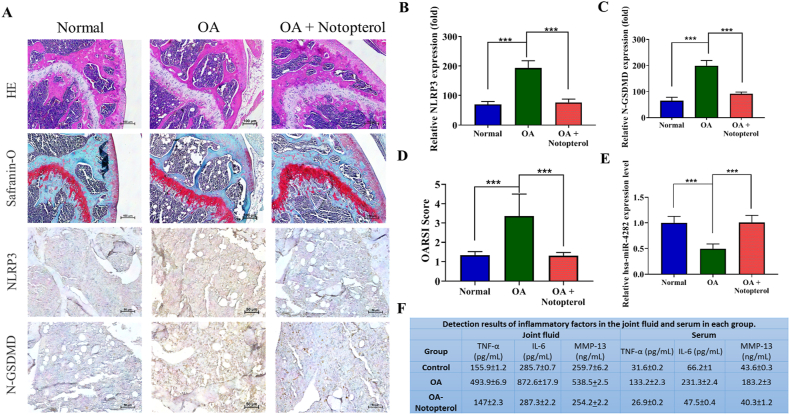
**Notopterol demonstrated a protective role in preventing OA deterioration in OA *in-vivo* mouse models.** (A) The representative keen joints section of OA mice demonstrated, the morphological changes and expression of Inflammasome and pyroptosis-associated marker expression. (B & C) The bar graph displays the quantified levels of inflammasome formation (NLRP3) and pyroptosis activity (N-GSDMD) markers following treatment with Notopterol, in comparison to the respective controls. (D) OARSI scoring. (E) qRT-PCR analysis of the expression profile of hsa-miR-4282 inflammatory and pro-inflammatory makers in Notopterol treatment and control group. (F) ELISA was performed to estimate the secretion of key inflammatory and protease makers in the presence and absence of Notopterol in the OA or sham group. ***P < 0.001.

After validating the *in-vitro* therapeutic effect of notopterol on the OA cells, we will establish its effectiveness for the OA treatment in *in-vivo*. The efficacy of Notopterol therapy was assessed using *in vivo* OA-mice animal model. The OARSI score has been applied to study the OA severity demonstrated in Supplementary Table S2. The cartilage in the OA group, when stained with Hematoxylin-Eosin (HE) and Safranin O-Fast Green, exhibited greater wear and a more pronounced decrease in thickness compared to the sham group. Additionally, the levels of inflammasome formation (NLRP3) and pyroptosis activity (N-GSDMD) markers were significantly higher in the OA group. (HE, Safranin-O, and IHC image as shown in Fig. 6A). However, after 8 weeks of continuous therapy with Notopterol in the OA + Notopterol group, the reduction in the expression level of NLRP3 and N-GSDMD was observed, demonstrating the protective role of Notopterol on the OA mice model, (quantification bar plot, Fig. 6B and C). which were statistically different from the findings in the OA group (***, p < 0.001). Our observations revealed that the average OARSI score for the control sham group indicated intact cartilage surfaces (Grade: 0), whereas the mice in the OA group exhibited destructed and eroded cartilage surfaces (Grade: 4). Furthermore, treatment with Notopterol was found to mitigate cartilage destruction in the OA + Notopterol group (Grade:2), as shown in Fig. 6D, with a statistically significant difference noted between the OA + Notopterol and OA groups (***, p < 0.001). This effect was associated with a decrease in the expression level of hsa-miR-4282 (Fig. 6E), highlighting its critical role in OA that is modulated by Notopterol treatment in a mouse model, as indicated in the quantification bar plot. Furthermore, the relative expression of NF-κB a major link between inflammation in tissue together with the expression of inflammatory and pro-inflammatory cytokine (TNF-α, IL-18, and IL-6) and protease expression (MMP13) genes demonstrated the Notopterol can effectively modulate the afore-mentioned markers (Fig. 6F). The inflammatory markers secretion in the OA + Notopterol group were comparable to those of the OA and sham groups in joint fluid and serum, the overall findings indicated that Notopterol can reverse the damage resulting from inflammasome and pyroptosis formation, as well as the Notopterol efficiently stimulates the expression hsa-miR-4282 expression in the treatment group results in the significant reduction of OA development.

## 4 Discussion

The initial stages of OA or the constraints of arthroplasty surgery, such as the possibility of adverse results and the short lifespan of prostheses are associated with morbidity. Therefore, the prevention of OA progression is crucial. Previous investigations have revealed that inflammation and ECM degradation play key roles in OA development. Notopterol, a compound derived from the *N. incisum* plant, has been proven to be enriched in patients treated with *N. incisum*. Furthermore, notopterol reduces inflammation by binding directly to three essential sites, in the JAK2/3 kinase domains, resulting in JAK-STAT signaling suppression in arthritis [8,10,29]. Thus, in the present study, we strived to elaborate potential of notopterol in reducing inflammation and pyroptosis in OA.

In this study, we have observed the safety of notopterol in the chondrocyte cell lines C204 and C28/12. Wang et al. found no evidence of toxicity caused by notopterol treatment, given that the levels of alanine aminotransferase, urea, aspartate aminotransferase, and CRE in notopterol-treated and control mice were not significantly different [29]. To induce inflammation in chondrocytes, we incubated the cells with IL-1β. Proinflammatory cytokines, particularly IL-1β and TNF-α, play a pivotal role in OA pathogenesis and cause articular cartilage degradation. The activation of catabolic processes that alter cartilage matrices appears to be one of the primary effects of IL-1β. Furthermore, IL-1β causes synoviocytes and chondrocytes to secrete degenerative enzymes such as MMPs. The cytokine receptors on articular chondrocyte surfaces are activated by IL-1β, which leads to the activation of the NF-κB signaling pathway [31,32]. This signaling pathway inhibits chondrocyte anabolic activity, either alone or in conjunction with other signaling pathways [33]. In this investigation, we found a dose-dependent link between notopterol and the production of the pro-inflammatory molecules IL-18 and TNF- in C204 and C28/12 cells, indicating the significance of notopterol in chondrocyte inflammation.

In OA joints, IL-1β can trigger apoptosis of chondrocytes [34]. Furthermore, when human chondrocytes from normal and OA cartilage were treated with human IL-1β, apoptosis increased [31]. Similarly, through DCF fluorescence, we showed that IL-1β incubation increased ROS production. As expected, notopterol decreased oxidative stress accumulation in C204 and C28/12 cells.

The earlier studies have indicated that notopterol can bind to the kinase sites of JAK2 [35], consistent with these findings, our present study demonstrated a similar effect, as the cells treated with IL-1 and notopterol demonstrated a dose-dependent decrease in JAK2 activity. Subsequently, we investigated the impact of notopterol-mediated attenuation of JAK2/STAT3 on cartilage degeneration. We investigated the expression patterns of MMP-13, a cartilage-degrading protease, as well as the cartilage matrix proteins Col II and aggrecan. Earlier research by Wang et al. suggested that notopterol binding requires three JAK2 binding sites at positions 931 (arginine), 980 (asparagine), and 981 (leucine). When these sites were altered in zebrafish, JAK2-3A led to reduced JAK2 and STAT5 phosphorylation, as well as decreased inflammatory processes [29]. The JAK-STAT signaling pathway has been implicated in the pathogenesis and progression of both rheumatoid arthritis (RA) and osteoarthritis (OA) in numerous studies [[36], [37], [38]]. It has also been identified as a critical target in various conditions, including cancer, autoimmune diseases like inflammatory bowel disease, ankylosing spondylitis, psoriatic arthritis, and psoriasis, as well as different forms of arthritis [39]. While inhibitors of the JAK/STAT signaling pathway have been developed and evaluated in clinical trials [40], concerns regarding their potential side effects persist. However, our findings demonstrate that notopterol treatment exhibited minimal toxicity, suggesting its potential as an effective inhibitor of inflammation.

Recently, researchers have discovered unique cell death mechanisms beyond the traditionally understood apoptosis and necrosis. These fresh modalities of cell death are often the outcome of extreme microenvironmental stressors. When pattern recognition receptors stimulate caspase-1 or caspase-11, the ensuing cascade leads to the release of pro-inflammatory cytokines such as interleukin-1 (IL-1) and interleukin-18 (IL-18) [41]. This series of events triggers pyroptosis, a highly regulated cell death process. Inflammasomes like NLRP3 activated through NF-κB signaling, play a critical role in the pathogenesis of low-grade inflammation [42]. They facilitate the maturation and release of IL-1 and IL-18, which are crucial mediators of inflammatory responses. The NLRP3 inflammasome complex is the most thoroughly researched among these structures. This complex includes NLRs (NLRP1, NLRP3, and NLRC4), the adapter protein ASC, pro-caspase-1, interferon-inducible protein 16, and pyrin. Together, these elements constitute a critical intersection between cell death and inflammation, furthering our understanding of disease mechanisms and potential therapeutic targets [[43], [44], [45]].

The transcription factor NF-κB has been identified as an important factor in the formation of inflammasomes and the initiation of pyroptosis, primarily through the stimulation of its downstream target, NLRP3. As of now, only a handful of studies have explored the potential role of the NLRP3 inflammasome in the pathogenesis of osteoarthritis (OA) [45]. Vandanmagsar and colleagues discovered that in a controlled animal model, the deletion of NLRP3 resulted in a reduction of obesity-triggered inflammasome activation within adipose tissues. In essence, obesity instigates pyroptosis through the activation of the NLRP3 inflammasome, a process that has previously been linked to the progression of osteoarthritis (OA) [46]. Upon activation by DAMPs or PAMPs, caspase-1 initiates inflammasome formation, such as that of NLRP3. In response, nearby macrophages in the cartilage initiate pyroptosis and release inflammasomes like NLRP3. This action enhances pyroptosis by increasing the release of IL-1 and IL-18 on cartilage surfaces, resulting in high concentrations of pro-inflammatory mediators in chondrocytes. Historically, it was believed that a positive feedback loop was formed by the stimulation of pyroptosis and the overexpression of IL-1β and IL-1-receptor I (IL-1RI) [47,48]. Contrary to Wang et al.'s findings, our results indicated that the administration of notopterol suppressed the NF-κB signaling pathway. This suppression, in turn, led to a reduction in NLRP3-mediated inflammasome generation and pyroptosis.

A multitude of naturally occurring substances, derived from plants (known as natural products), have been found capable of altering the expression of multiple miRNAs. These miRNAs, in turn, serve diverse roles in biological processes [49]. miRNAs, noncoding RNA molecules approximately 22 nucleotides long, regulate gene expression post-transcriptionally. Highly conserved, these miRNAs bind to the 3′ UTR of target mRNAs either imperfectly or perfectly, leading to mRNA degradation or protein translation suppression. Thousands of these miRNAs, prevalent in both plants and animals, have been identified. It is understood that they modulate the expression of more than 30% of proteins engaged in vital biological functions, such as cell development, differentiation, proliferation, and apoptosis [50].

In this study, we demonstrated the potential of notopterol to alter miRNA expression, specifically mir-4282. Using the TargetScan database, we identified a set of miRNAs that could potentially target NF-κB and NLRP3. Post notopterol treatment, we scrutinized these miRNAs' expression in chondrocytes. Our findings indicate an increased miR-4248 expression due to notopterol. By employing an inhibitor and mimic of miR-4248, we validated that this miRNA could potentially influence the expression of its perceived targets, NF-κB and NLRP3. This suggests that notopterol may suppress the expression of NF-κB and NLRP3 in a manner akin to the miR-4248 mimic. Research on the role of miR-4282 in diseases remains scarce. However, based on bioinformatics analysis, RT-qPCR, and Western blotting results, Myc may be the predictive target gene of miR-4282 in breast cancer. By modulating Myc, miR-4282 appears to mitigate the incidence and progression of this disease. Another study found that miR-4282 impeded tumor growth in oral squamous cell carcinoma cells by targeting LIN28B and downregulating ZBTB2, suggesting its potential as a novel biomarker for OSCC. Similarly, Kang et al. found miR-4282 to function as a tumor suppressor in colorectal cancer cells, targeting Sema3E, and modulating its mRNA degradation to curb tumor cell growth [51,52].

## 5 Conclusion

**Fig. 7:**
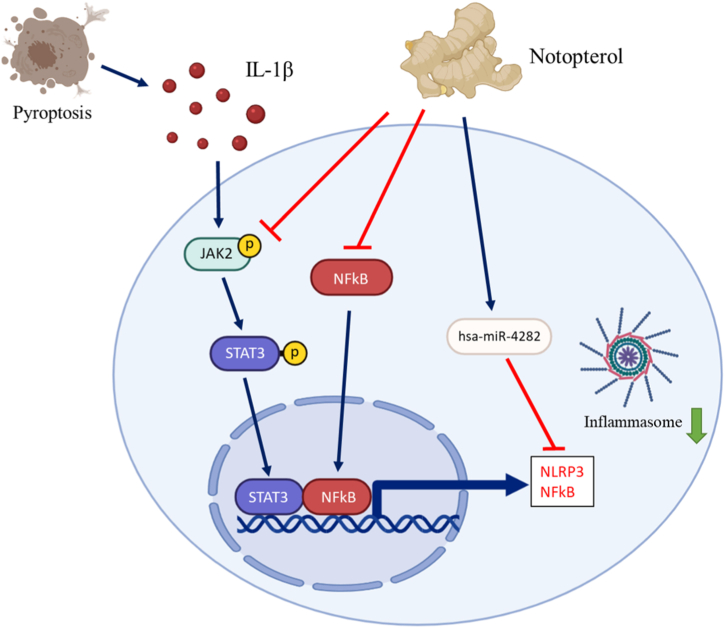
Schematic abstract illustrating Notopterol's effect against the formation of pyroptosis and inflammasome through the mechanistic inhibition of the JAK2/STAT3 pathway and has-miR-4282 induction to inhibit its target genes NF-κB and NLRP3.

As per our knowledge, this study illustrates that notopterol may confer protective properties to chondrocytes. It does this by inhibiting inflammasome formation and pyroptosis via manipulation of the JAK2/STAT3 and miR-4282/NF-κB/NLRP3 signaling pathways (Fig. 7). However, the limited efficacy of Notopterol highlights the need for a more thorough exploration in future research, especially regarding its clinical relevance and potential use as part of a combined therapy approach for osteoarthritis treatment. The comprehensive understanding of notopterol's pharmacodynamics and pharmacokinetics in the treatment of osteoarthritis (OA) necessitates further research. Consequently, our findings suggest that notopterol holds promise as a potentially potent natural compound in the therapeutic arsenal against OA.

## Fundings

This research received financial support from the Ministry of Science and Technology (grant number MOST109-2410-H038-015 and MOST 110-2410-H-038-014 ), awarded to Yen-Shuo Chiu.

## Ethics approval and consent to participate

The Laboratory Animal Committee of 10.13039/501100004700Taipei Medical University granted ethical approval for this study under protocol number LAC-2020-0146.

## Consent for the publication

The authors assert that they have no financial or non-financial interests that could be influenced by the publication of this manuscript, either now or in the future.

## Data and materials availability

The dataset utilized and analyzed in this study are available to the public as detailed within the manuscript.

## CRediT authorship contribution statement

**Ko-Ta Chen:** Writing – review & editing, Writing – original draft, Conceptualization. **Chi-Tai Yeh:** Writing – review & editing, Writing – original draft, Supervision, Project administration. **Vijesh Kumar Yadav:** Formal analysis, Data curation, Conceptualization. **Narpati Wesa Pikatan:** Resources, Methodology, Investigation. **Iat-Hang Fong:** Software, Resources, Methodology, Investigation. **Wei-Hwa Lee:** Visualization, Validation, Resources. **Yen-Shuo Chiu:** Writing – review & editing, Supervision, Funding acquisition, Conceptualization.

## Declaration of competing interest

The authors assert that they have no financial or non-financial interests that could be influenced by the publication of this manuscript, either now or in the future.
