# Supplementary material for: Notopterol mitigates IL-1β-triggered pyroptosis by blocking NLRP3 inflammasome via the JAK2/NF-kB/hsa-miR-4282 route in osteoarthritis
Source: Heliyon. 2024 Mar 13;10(6):e28094. doi: 10.1016/j.heliyon.2024.e28094 (PMC10963379; doi:10.1016/j.heliyon.2024.e28094)
Supplement: Multimedia component 1 [file mmc1.docx]

**Supplementary Information**

**Notopterol Mitigates IL-1β-Triggered Pyroptosis by Blocking NLRP3 Inflammasome via the JAK2/NF-κB/miR-4282 Route in Osteoarthritis**

Ko-Ta Chen^1#^, Chi-Tai Yeh^2,3^**^#^**, Narpati Wesa Pikatan^2^, Vijesh Kumar Yadav^2^, Iat-Hang Fong^2^, Wei-Hwa Lee^4^, Yen-Shuo Chiu^5,6,7*^

^1^ Department of Orthopedics, Taipei Medical University Hospital, Taipei 11031, Taiwan;

^2^ Department of Medical Research & Education, Taipei Medical University - Shuang Ho Hospital, New Taipei City 23561, Taiwan.

^3^ Continuing Education Program of Food Biotechnology Applications, College of Science and Engineering, National Taitung University, Taitung 95092, Taiwan

^4^ Department of Pathology, Taipei Medical University-Shuang Ho Hospital, New Taipei City, Taiwan

^5^ Department of Orthopedics, Shuang Ho Hospital, Taipei Medical University, Taipei 23561, Taiwan

^6^ School of Nutrition and Health Sciences, College of Nutrition, Taipei Medical University, Taipei 11031, Taiwan

^7^ Research Center of Geriatric Nutrition, College of Nutrition, Taipei Medical University, Taipei 11031, Taiwan

# Equal contribution

*Author to whom correspondence should be addressed.

Yen-Shuo Chiu, MD., PhD

Department of Orthopedics, Shuang Ho Hospital, Taipei Medical University, Taipei 23561, Taiwan. Tel: +886-2-2490088 ext. 8881, Fax: +886-2-2248-0900. E-mail: [g556096005@tmu.edu.tw](mailto:g556096005@tmu.edu.tw)

**Supplementary Table S1.** The membranes were incubated in primary antibodies.

| **No.** | **Target** | **Dilution** |  | **Source** | |
| --- | --- | --- | --- | --- | --- |
| 1 | p-JAK2 | 1:1000 | 125 | #3771 | Cell Signaling |
| 2 | JAK2 | 1:1000 | 125 | #3230 | Cell Signaling |
| 3 | p-STAT3 | 1:1000 | 86 | #9145 | Cell Signaling |
| 4 | STAT3 | 1:1000 | 86 | #9139 | Cell Signaling |
| 5 | NFκB | 1:1000 | 65 | #8242 | Cell Signaling |
| 6 | MMP13 | 1:1000 | 60 | #69926 | Cell Signaling |
| 7 | Coll II | 1:1000 | 141 | ab34712 | Abcam |
| 8 | Aggrecan | 1:1000 | 250 | ab3778 | Abcam |
| 9 | NLRP3 | 1:1000 | 118 | ab263899 | Abcam |
| 10 | Cas-1 | 1:1000 | 29 | ab207802 | Abcam |
| 11 | ASC | 1:1000 | 22 | ab180799 | Abcam |
| 12 | N-GSDMD | 1:1000 | 31 | ab215203 | Abcam |
| 13 | IL-18 | 1:1000 | 22 | ab243091 | Abcam |
| 15 | β-actin | 1:10000 | 42 | 66009-1-Ig | proteintect |

**Supplementary Table S2:** OARSI osteoarthritis cartilage histopathology grading for histopathological changes in OA tissues after Notopterol treatment.

|  | | | | |
| --- | --- | --- | --- | --- |
| ***Observer 1*** | ***Observer 2*** | ***Average***  ***grading (Score*)*** | |  |
| Normal control | 0 | 0 | G 0 | |
| Osteoarthritis (OA) | 4 | 4 | Grade 4 | |
| 1.5 | 2.5 | Grade 2 | |  |
| ***Stage % Involvement (surface, area, volume)*** | | | |  |
|  | ***Stage 1 (<10%)*** | ***Stage 2 (10–25%)*** | ***Stage 3***  ***(25–50%)*** | ***Stage 4 (>50%)*** |
| Grade 1 (surface intact) | 1 | 2 | 3 | 4 |
| Grade 2 (surface discontinuity) | 2 | 4 | 6 | 8 |
| Grade 3 (vertical fissures, clefts) | 3 | 6 | 9 | 12 |
| Grade 4 (erosion) | 4 | 8 | 12 | 16 |
| Grade 5 (denudation) | 5 | 10 | 15 | 20 |
| Grade 6 (deformation) | 6 | 12 | 18 | 24 |


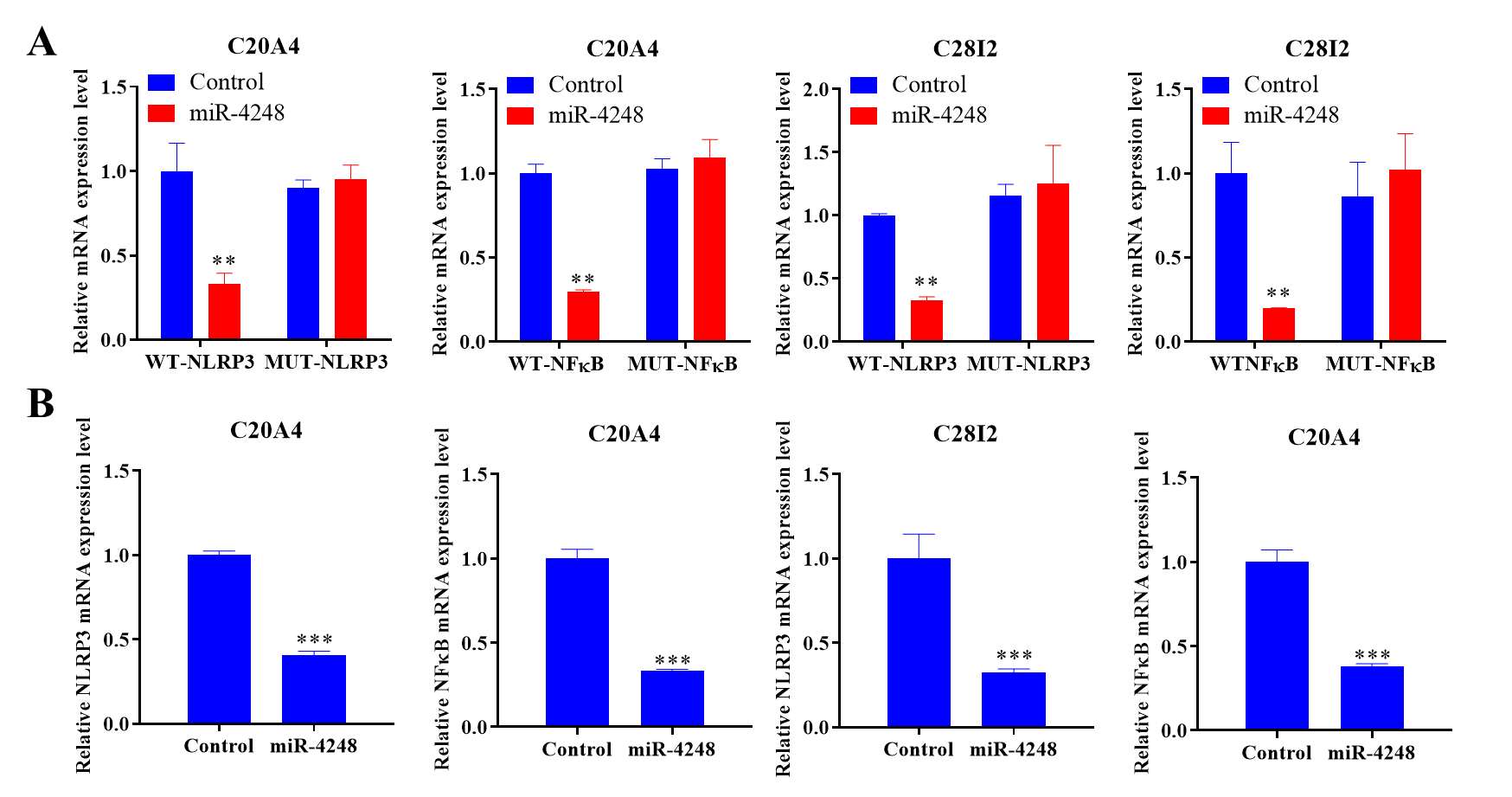


**Supplementary Figure S1: miR‐4248 targeted NLRP3 and NF-κB in vitro.** The luciferase

reporter constructs containing the wild‐type (WT‐NLRP3 or WT-NF-κB) or mutant (MUT‐NLRP3 or MUT-NF-κB) sequence were transfected in C20A4 or C28/12 cells. (A) WT‐NLRP3 and WT-NF-κB or MUT‐NLRP3 and MUT-NF-κB were cotransfected into cells with miR‐4248 mimics or their corresponding negative controls. (B) mRNA level of NLRP3 and NF-κB in cells transfected with miR‐4248 mimics. Error bars stand for the mean ± SD of at least triplicate experiments. mRNA, messenger RNA; SD, standard deviation *p < 0.05; ***p < 0.001
