# Supplementary material for: Notopterol mitigates IL-1β-triggered pyroptosis by blocking NLRP3 inflammasome via the JAK2/NF-kB/hsa-miR-4282 route in osteoarthritis
Source: Heliyon. 2024 Mar 13;10(6):e28094. doi: 10.1016/j.heliyon.2024.e28094 (PMC10963379; doi:10.1016/j.heliyon.2024.e28094)
Supplement: Multimedia component 2 [file mmc2.docx]

**Western Blot Raw Data**

**Notopterol Mitigates IL-1β-Triggered Pyroptosis by Blocking NLRP3 Inflammasome via the JAK2/NF-κB/miR-4282 Route in Osteoarthritis**

Ko-Ta Chen^1#^, Chi-Tai Yeh^2,3^**^#^**, Narpati Wesa Pikatan^2^, Vijesh Kumar Yadav^2^, Iat-Hang Fong^2^, Wei-Hwa Lee^4^, Yen-Shuo Chiu^5,6,7*^

^1^ Department of Orthopedics, Taipei Medical University Hospital, Taipei 11031, Taiwan;

^2^ Department of Medical Research & Education, Taipei Medical University - Shuang Ho Hospital, New Taipei City 23561, Taiwan.

^3^ Continuing Education Program of Food Biotechnology Applications, College of Science and Engineering, National Taitung University, Taitung 95092, Taiwan

^4^ Department of Pathology, Taipei Medical University-Shuang Ho Hospital, New Taipei City, Taiwan

^5^ Department of Orthopedics, Shuang Ho Hospital, Taipei Medical University, Taipei 23561, Taiwan

^6^ School of Nutrition and Health Sciences, College of Nutrition, Taipei Medical University, Taipei 11031, Taiwan

^7^ Research Center of Geriatric Nutrition, College of Nutrition, Taipei Medical University, Taipei 11031, Taiwan

# Equal contribution

*Author to whom correspondence should be addressed.

Yen-Shuo Chiu, MD., PhD

Department of Orthopedics, Shuang Ho Hospital, Taipei Medical University, Taipei 23561, Taiwan. Tel: +886-2-2490088 ext. 8881, Fax: +886-2-2248-0900. E-mail: [g556096005@tmu.edu.tw](mailto:g556096005@tmu.edu.tw)

**
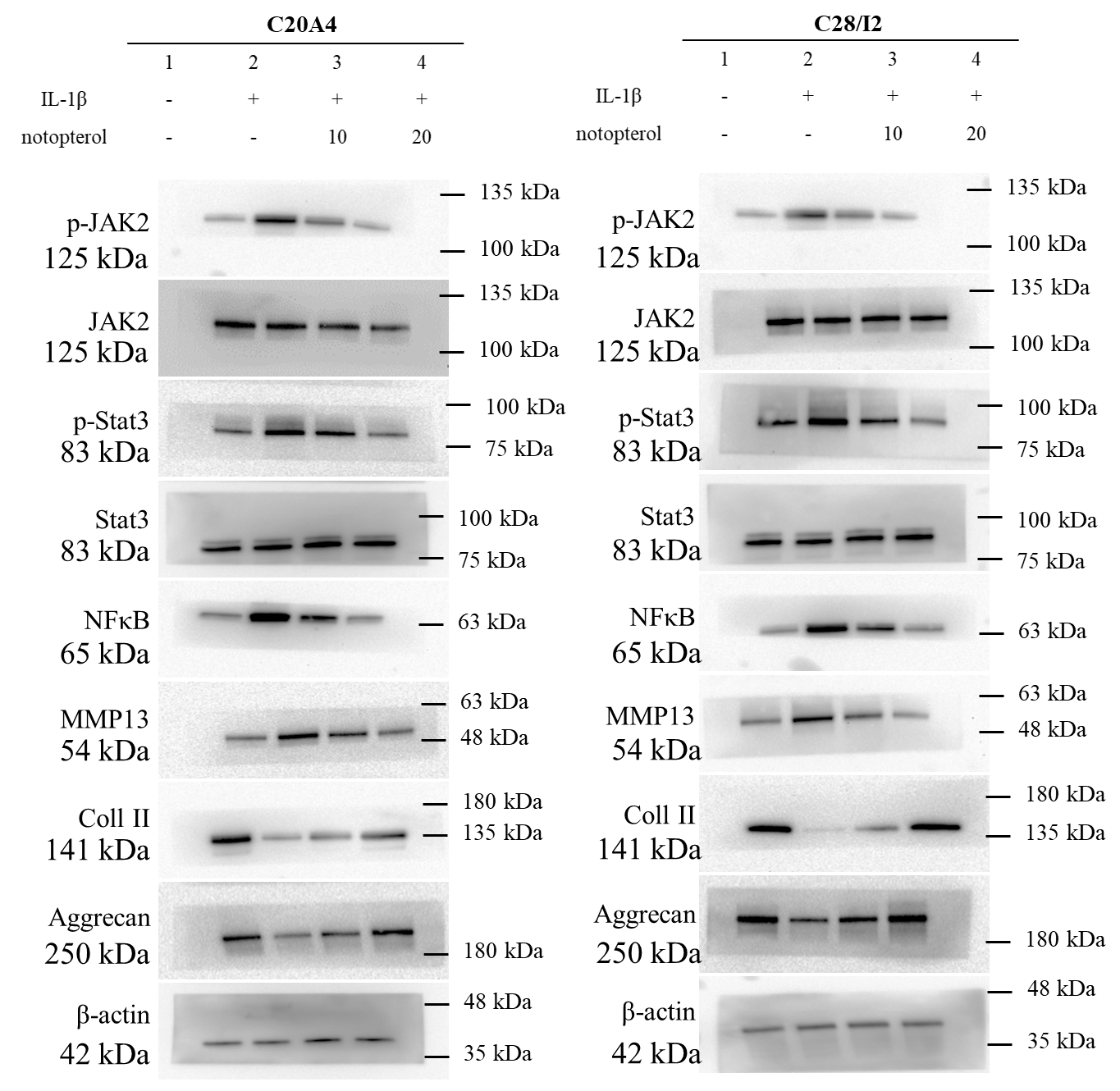
**

**Supplementary Western Blot Raw Data S1.** Full-size blots of Figure 3A, B


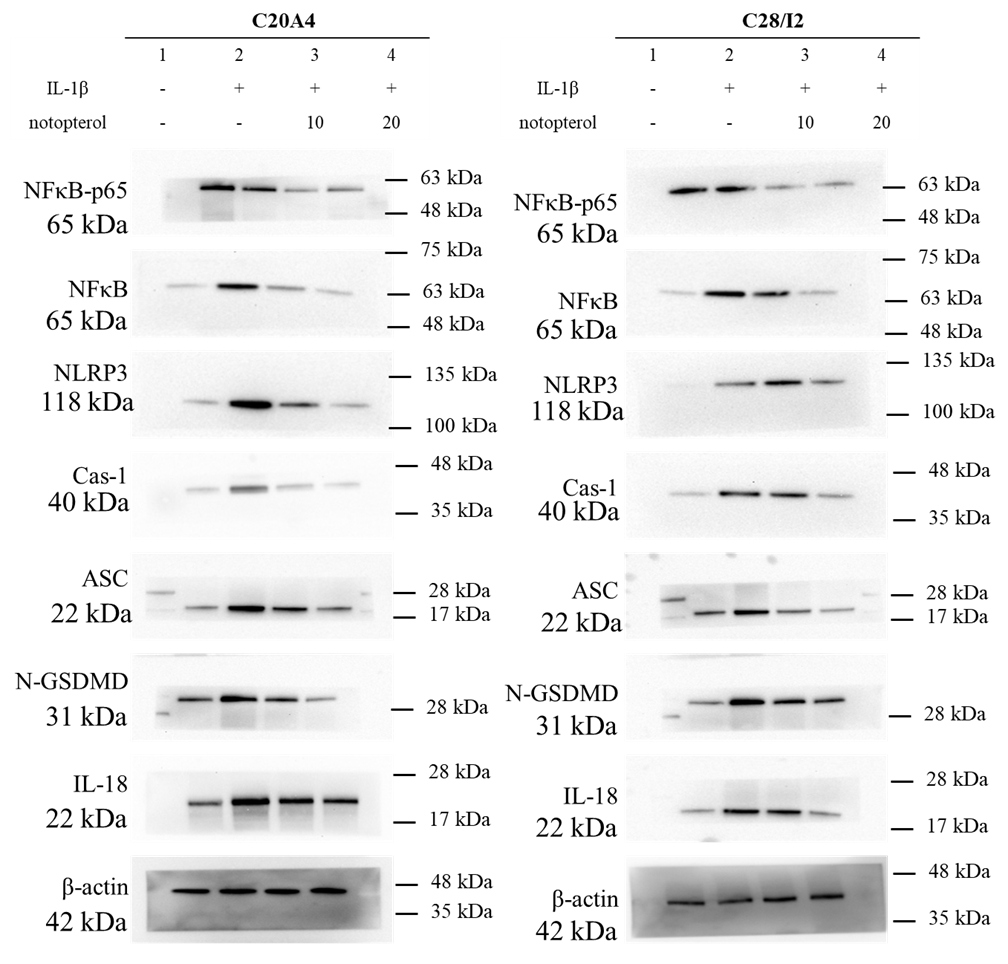


**Supplementary Western Blot Raw Data S2.** Full-size blots of Figure 4A, B


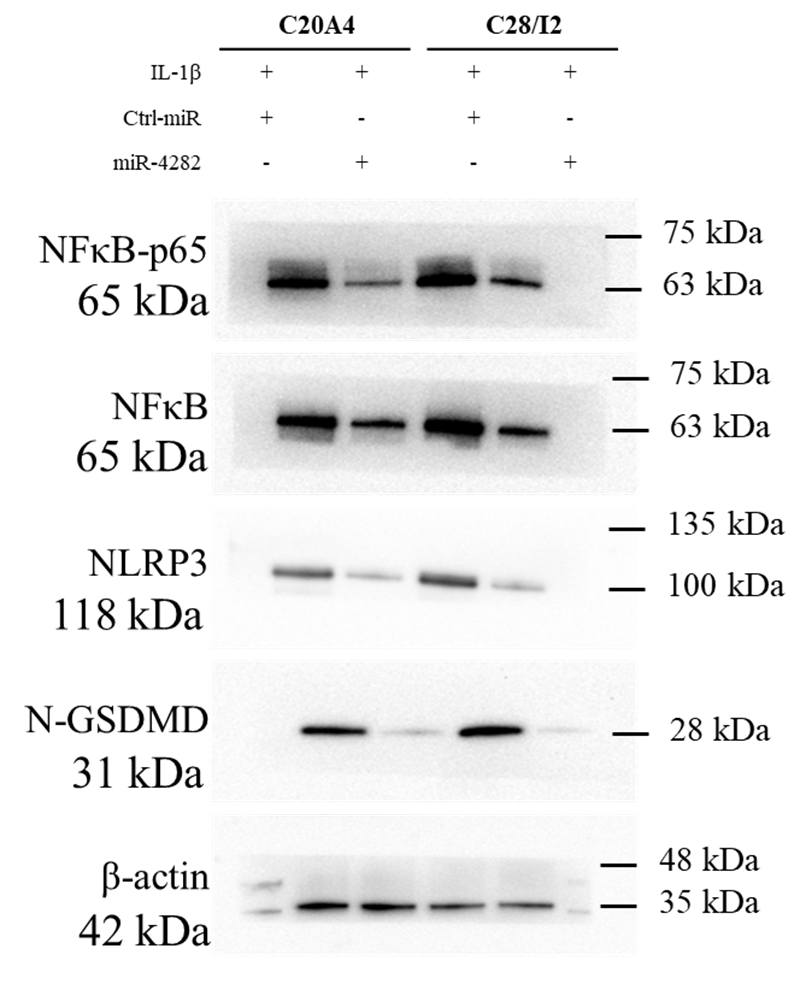


**Supplementary Western Blot Raw Data S3.** Full-size blots of Figure 5F
